# Conditional cash transfers to prevent mother-to-child transmission in low facility-delivery settings: evidence from a randomised controlled trial in Nigeria

**DOI:** 10.1186/s12884-019-2172-3

**Published:** 2019-01-16

**Authors:** Jenny X. Liu, Jennifer Shen, Nicholas Wilson, Svetha Janumpalli, Patrick Stadler, Nancy Padian

**Affiliations:** 1 Department of Social and Behavioral Sciences, Institute for Health & Aging, University of California, San Francisco, 3333 California Street, Suite 340, San Francisco, CA 94118 USA; 2 Institute for Health Policy Studies, University of California, San Francisco, 3333 California Street, Suite 265, San Francisco, CA 94118 USA; 3 Office of Evaluation Sciences and Department of Economics, Reed College, 3203 SE Woodstock Blvd, Portland, OR 97202 USA; 4 New Incentives, Walnut Creek, California USA; 5 School of Public Health, University of California, Berkeley, 770 University Hall, Berkeley, CA 94720-7360 USA

**Keywords:** Prevention of mother-to-child transmission (PMTCT), Conditional cash transfers (CCT), Nigeria, HIV/AIDS, Facility delivery

## Abstract

**Background:**

Nigeria suffers from the highest burden of mother-to-child transmission worldwide. To increase retention in care and prevention programmes, we piloted and evaluated a conditional cash transfer (CCT) programme for preventing mother-to-child transmission (PMTCT) in Akwa Ibom, Nigeria.

**Methods:**

In a randomised controlled trial, pregnant women testing positive for HIV during antenatal care registration at three public hospitals were randomised to one of two study arms: (1) offered enrolment into the CCT programme or (2) continue in standard care for (PMTCT). In the CCT programme, women could receive a compensation package totaling 33,300 Naira (~US$114) for enroling, delivering at the facility, and obtaining a newborn early infant diagnosis (EID) test. The intent-to-treat (ITT) and per protocol (PP) effects of the programme on the primary outcomes of facility delivery and EID testing and on the secondary outcome of nevirapine administration were estimated with logistic regressions.

**Results:**

From August 1, 2015 to April 19, 2017, 554 pregnant women tested positive for HIV; 273 were randomised to standard care and 281 were offered enrolment into the CCT intervention. Women offered the CCT programme were more likely to give birth at the facility (*n* = 109/263; 41.4%) compared to women in standard care (*n* = 80/254; 31.5%), an absolute difference of 9.9% (OR = 1.54, 95% CI: 1.07–2.21, *p* = 0.019). For EID testing there was an absolute difference of 12.8% between those offered the CCT intervention (*n* = 69/263; 26.2%) and those in standard care (*n* = 34/254; 13.4%; OR = 2.30, 95% CI 1.46–3.62, *p* = 0.000). PP results show larger differences for both facility deliveries (16.7% absolute difference; OR = 2.02, 95% CI 1.38–2.98, *p* = 0.000) and EID testing (18.9% absolute difference; OR = 3.09, 95% CI 1.93–4.94, *p* = 0.000) among intervention enrolees. Over 86% of the facility-delivered newborns received nevirapine, and ITT and PP estimates were similar to those for facility deliveries.

**Conclusions:**

Results show that CCTs improved the likelihood of HIV-positive women giving birth at a facility, of nevirapine being administered to their newborn, and of undergoing EID testing in Akwa Ibom, Nigeria. Effects are especially large among those who agreed to participate in the CCT intervention.

**Trial registration:**

ClinicalTrials.gov NCT02447159, May 18, 2015.

## Background

Nigeria has one of the highest infant mortality rates in the world and, together with India, Democratic Republic of the Congo, Pakistan, and China, accounts for half of under-five child mortality worldwide [1]. HIV continues to be one of the main causes of mortality and morbidity in Nigeria [2], and children in Nigeria are disproportionately affected—1 in 3 children with HIV worldwide are born in Nigeria [3].

HIV transmission from mother to child is preventable, but requires adherence to the many steps involved in the PMTCT cascade, including initiating antenatal care, HIV testing and result retrieval, adhering to antiretroviral (ARV) treatment, adhering to infant treatment including nevirapine administration at birth, and newborn follow-up testing and care [4]. With timely access to antiretrovirals, HIV-positive pregnant women can improve their own health while providing prophylaxis to prevent HIV transmission during pregnancy and ultimately reduce rates of transmission from mother to child to less than 5% [2, 5]. However, Nigeria continues to struggle with high unmet need for PMTCT; of the over 192,000 infected pregnant women in 2013, fewer than 58,000 received some PMTCT services, resulting in a coverage rate of only 30% [6].

Similar to many countries with generalized HIV epidemics, pregnant women in Nigeria are lost at each step along the PMTCT cascade. Barriers to optimal utilization of PMTCT services exist in both demand and supply. The quality of care at facilities may be poor, including stigmatizing attitudes and poor training among staff [7], inadequate voluntary counseling [8], drug stockouts [7], and payments (both formal and informal) for services [7]. In Nigeria, the costs of transportation to the clinic, registration, medications, and laboratory tests are especially burdensome for impoverished patients [9]. In our study, women delivering at public facilities spent an average of 16,000 Naira (~US$55), or 2.2% of annual per capita income [10], with some spending upwards of 70,000 Naira (~US$233) or 9.5% of annual per capita income. Additionally, woman may fear involuntary HIV disclosure and associated negative community reactions due to prevailing social norms, including challenges engaging men in prevention and lack of knowledge about appropriate care and treatment [11–15].

To improve the uptake of PMTCT services, the Government of Nigeria has implemented PMTCT programmes in over 5700 facilities nationwide, trained thousands of birth attendants, and has begun free distribution of ARV drugs [9]. Yet, despite improvements in the provision of PMTCT services, uptake remains poor. Only 30% of pregnant women living with HIV in 2013 received ARV treatment to prevent the transmission of HIV to their child, and only 2.3% of infants born to HIV-infected mothers received ARV prophylaxis [16]. One major issue is the low incidence of giving birth at a health facility where HIV-exposed newborn infants are routinely administered nevirapine, a critical step in the PMTCT cascade [17]. In 2013, fewer than 2 out of every 5 births were delivered in a health facility [18]. While HIV and PMTCT services may ostensibly be free at public faciltiies, other services such as facility delivery, and laboratory tests may not be.

Interventions using cash incentives have proven effective for a variety of behaviors related to HIV prevention, including voluntary medical male circumcision [19–21], adherence to antiretroviral therapy, retrieval of HIV test results, and making better choices in the number of sexual partners [22, 23]. Similarly, cash incentives can reduce barriers to maternal and newborn health service utilization; they not only enable beneficiaries to overcome short- and long-term financial barriers (e.g., transportation, service fees), but also can counter social stigma preventing HIV-positive women from seeking services (insofar as the public reason for the visit can be to obtain funds) [24]. Because cash incentives address multiple demand-side barriers, they are an especially promising tool for increasing PMTCT service utilization. Cash incentives may also leverage present bias tendencies by rewarding immediate uptake of highly beneficial services that lead to larger future benefits, such as having a child grow up HIV-free [25, 26].

In collaboration with the State Ministry of Health, New Incentives, a non-governmental organization (NGO) designed and implemented a CCT intervention in Akwa Ibom, Nigeria in June 2014 to improve utilization of health services for PMTCT. Located in the Niger Delta, Akwa Ibom has one of the highest HIV prevalence rates in Nigeria [27]. As a PMTCT priority state, hundreds of PMTCT sites had been activated, but remained underutilized [11, 27]. In the intervention, HIV-positive pregnant women presenting for antenatal care (ANC) were given incentives conditional on completing steps on the PMTCT treatment cascade. As part of the evalution of the intervention, women were randomly assigned to be offered enrolment into the CCT programme or continue in standard PMTCT care. This paper reports on the effects of the CCT intervention on the primary outcomes of facility delivery and EID testing and on the secondary outcome of nevirapine administration.

## Methods

### Intervention

HIV-positive women registering for ANC were eligible to receive up to 3 transfers during their pregnancy through 10 weeks after birth for achieving milestones that are summarized in Table 1: 7000 Naira (~US$24) after ANC registration plus 300 Naira (~US$1) of mobile phone credits (“Transfer 1”); 20,000 Naira (~US$70) when the participant gave birth at the same health facility where she registered for ANC (“Transfer 2”); and 6000 Naira (~US$20) when she returned to the facility to obtain an early infant diagnosis (EID) test for HIV (“Transfer 3”). Transfer amounts were determined based on stakeholder discussions with local PMTCT technical working groups, and on the amount of formal and informal fees charged by public facilities in the state. Facility delivery was determined to be the most important step in the PMTCT cascae within the local context, and thus was assigned the highest transfer amount. The total compensation was valued at 33,300 Naira (~US$114).

**Table 1:** Cash transfer conditions and payouts

Transfer	Condition	Amount
1	Enrol in the CCT programme^a^	Part 1: 1000 Naira (~US$3) + 300 Naira (~US$1) of mobile phone credits Part 2: 6000 Naira (~US$20)
2	Deliver at the facility where enroled	20,000 Naira (~US$70)
3	Obtain an EID test (only eligible to women who delivered at the facility)	6000 Naira (~US$20)

^a^Part 1 of Transfer 1 was given at the time of enrolment. Part 2 of Transfer 1 was available for retrieval at a bank 1 day after enrolment

For each transfer, after verifying step completion against hospital records, cash was disbursed via digital money tokens where beneficiaries received a secured code by text message that could be redeemed at a participating ATM or bank. Transfer 1 was given in two parts. An initial cash transfer of 1000 Naira and 300 Naira of mobile phone credits were given at the time of enrolment to address women’s concerns that the intervention was fake and to overcome barriers in retrieving the remaining portion of Transfer 1 (6000 Naira) from a bank the next business day. These costs mainly covered transportation for getting to the bank and having enough phone credits to call the intervention officer afterward to verify receipt of cash.^1^ In addition to cash payouts, CCT intervention participants received seven short phone calls and text messages spaced throughout the intervention to verify that conditions were met and cash was disbursed, but which also included messages reinforcing the importance of delivering at a facility and obtaining a newborn EID test.

### Evaluation study design

We conducted a individual randomised controlled trial at 3 public, secondary-level health facilities in Akwa Ibom State, Nigeria, that had the highest HIV burden and that provided comprehensive PMTCT services and emergency obstetric care. These facilities were also selected to represent different clientele populations, from relatively poorer and more rural populations to more well-off and urban populations. The institutional review boards (IRBs) at the Akwa Ibom State Ministry of Health, Reed College, and the University of California, San Francisco approved the study protocol and procedures.

### Outcomes

The primary outcomes for the study were:

1. The percentage of pregnant women who delivered their baby at the facility in which they were first enroled for ANC.
2. The percentage of mothers who obtained an early infant diagnosis testing 6–8 weeks after giving birth to their child at the facility in which they were first enroled for ANC.

Data for these primary outcomes were obtained via weekly abstraction of patient records from various hospital registers by intervention officers.^2^ As EID testing should take place 6–8 weeks after birth, any testing up to 10 weeks post-birth was counted to allow sufficient time for women to present at the facility.

Within the CCT intervention arm, differences across 3 subgroups were also compared: (1) whether the participant was newly diagnosed with HIV on the day of ANC registration or diagnosed at a prior time; (2) whether the participant had been pregnant before or the current pregnancy was her first; and (3) among women with prior pregnancies, whether the participant delivered her last child at a facility or elsewhere. The dimensions were chosen based on *a priori* hypotheses about factors likely to moderate the effect of the intervention. For example, participants newly diagnosed on the day of ANC registration may not have had time to fully comprehend their HIV status (i.e. initial non-acceptance of their status) [28], the short- and longer-run impacts of HIV on their life, and the importance of PMTCT for her own health and that of her baby [29, 30]. Similarly, mothers with prior pregnancy experience may better understand the importance of ante-, peri-, and post-natal care and be more likely to utilize health services compared to those pregnant for the first time. Lastly, women who previously gave birth at a facility may be more apt to do so again.

When the evaluation was originally designed, two secondary outcomes were specified: (1) percentage of pregnant women who picked up their ARV drugs at the facility at least once between enroling in ANC and giving birth to their baby; and (2) percentage of mothers who picked up nevirapine for their newborn infant at the facility after giving birth. We could not analyze the first outcome because hospital records on ARV drug pickups for mothers were not consistently kept across all three facilities and could not be verified with the same degree of certainty and accuracy as the other outcomes. The second outcome of nevirapine administration was chosen because this step, although routine at all facilities, may not occur due to unintended hospital errors (e.g., miscommunication, errors in medical records, timing of patient discharge).

### Participants

To be eligible for the study, women had to be HIV-positive, pregnant, attend ANC at 1 of the 3 participating study facilities, and not previously participated in a CCT programme administered by the implementing NGO.^3^ Women who were found to be HIV-negative or not pregnant, including those testing negative when randomly chosen for confirmatory HIV and pregnancy testing during enrolment into the CCT programme,^4^ were excluded from the study. In addition, biometric verification (via facial recognition) for all women enroling in the CCT programme was conducted to identify women who had previously participated in another CCT programme implemented by the same NGO.

At each participating study facility, per facility standard operating procedures, HIV testing was routinely conducted on all women registering for ANC care (HIV rapid test followed by 2 routine confirmatory tests the same day). One day of the week at each facility was designated as ANC registration day. As HIV testing was completed among ANC attendees, a routine step in ANC registration, the clinic’s HIV testing officer wrote the names and phone numbers of pregnant women who tested positive for HIV on a list, which was then given to the CCT intervention officer stationed in a separate room (to preserve privacy and confidentiality). Pregnant women coming for ANC on a non-ANC registration day were instructed by facility staff to return on an ANC registration day for HIV testing and initation of care. The CCT intervention officer then entered each patient’s identification and phone number into an electronic database via a phone-based application (i.e. doForms), which then revealed the study arm assignment for each individual according to a pre-determined, computer-generated random assignment list developed by the first author (who did not have any direct patient contact during the study) prior to the start of study recruitment. Because basic patient information (i.e. age, estimated due date) and primary outcomes data for this study (i.e. facility delivery, EID) were extracted from hospital records, informed consent for the control condition or for those who refused to participate in the intervention (see below) was deemed unnecessary by the IRBs as all analyzed data were de-identified once study recruitment ended. To minimize any undue bias in care provided to study participants, the randomised assignment was masked to all clinicians at study sites.

After obtaining the randomisation assignment, the intervention officer invited women assigned to the CCT arm to speak privately about the opportunity to enrol in the programme. If the participant expressed interest, she was first screened for eligibility. Women found to be ineligible were immediately thanked for their interest and removed from the intervention and study. For eligible women, the intervention officer (bilingual in English and Ibibio, the local ethnic language) explained the CCT programme following a standardized script. In addition to the steps required to retrieve the cash transfers, women in the intervention were asked to respond to additional survey questions (at enrolment and in follow-up phone calls) on their socio-demographic background and spending and health behaviors during the intervention period. After giving written informed consent, these women's names, ages, and estimated due dates were verified against their patient cards, and then given a participant identification number and a CCT programme card that tracked progress for each incentivized step. Women randomised to the CCT intervention but who refused to enrol in the intervention (*n* = 68) were still tracked over time by the study for the intent-to-treat (ITT) analysis.

Women assigned to the control group did not engage further with the intervention officer and simply received routine care at the ANC clinic. At the end of each ANC registration day, the intervention officer retrieved each control woman’s hospital patient card to verify eligibility and to extract her age and estimated due date, the only two background characteristics that were routinely recorded for all patients on hospital registers. Any woman discovered to be ineligible based on information from the patient card and CCT intervention records was removed from the study.

To retrieve data for the delivery, nevirapine administration, and EID testing outcomes, intervention officers regularly checked hospital registers in different wards to identify RCT participants among all patient records. Strict procedures were followed to ensure that records from different registers were for the same individual in instances where patient identification numbers or names may have be written incorrectly. In cases where it was unclear if a women who delivered in a facility or completed an EID test for her infant was a study participant, additional patient data given during ANC registration (e.g., medical and birth history, village name, tribe, religion or denomination, partner information, phone numbers) were used to help verify her identity.

### Statistical analysis

Sample sizes were calculated using the DS Research online power calculator.^5^ Assuming a 20 percentage point increase in facility delivery from 50% without the CCT programme (i.e. giving the largest possible variance) to 70% with the CCT programme, a sample size of 500 women (250 in each arm) was calculated to have 99.9% power (two-tailed test). An additional 10% upward adjustment was made to account for refusals to participate and any loss to follow-up, bringing the total target sample size to 550.

To assess the comparability of our study arms, we compared the available baseline characteristics (i.e. recruitment site, age, and month of pregnancy at ANC enrolment) of participants between study arms.^6^ We conducted logistic regression analyses to estimate the ITT and per protocol (PP) main effects of the programme. We estimated both the unadjusted and adjusted (i.e. controlling for recruitment site, the only unbalanced characteristic) effect estimates and reported the risk differences and odds ratios. To estimate PP effects, we restricted our sample to women who agreed to enrol in the CCT programme and conducted the same regression analyses. Subgroup analyses by parity, place of delivery for the last birth, and time of HIV diagnosis were conducted only for the subset of women randomised to the CCT intervention arm and who agreed to enroll in the CCT programme.

Statistical analyses were performed using Stata/MP 13.1. The funder of the study did not have any role in the study design, data collection, data analysis, interpretation, or writing of the results.

## Results

**Fig. 1:**
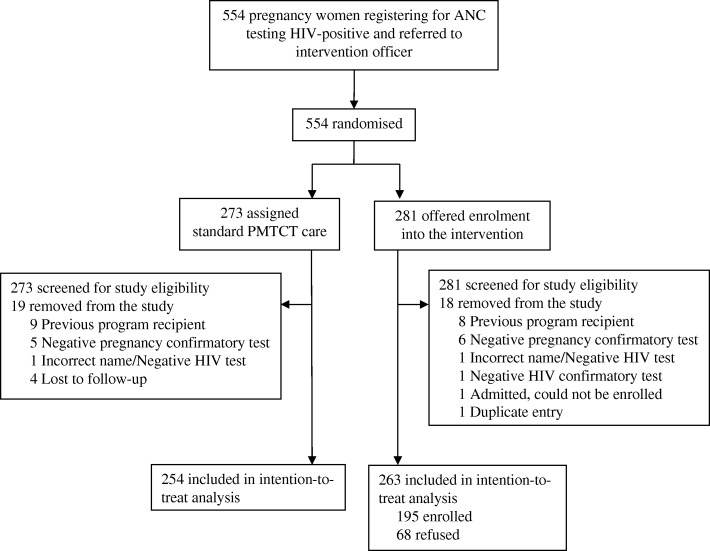
Trial profile

From August 1, 2015 to May 9, 2016, 554 pregnant women were eligible for inclusion into the trial during initial screening, enroled into the study, and were randomised to a study arm: 273 were assigned to standard care and 281 were assigned the CCT intervention. Figure 1 displays the trial profile. During regular record abstraction for women in the standard care arm, 19 were removed from the study after being found ineligible for having already been a beneficiary for another CCT programme run by the same NGO or was confirmed not to be pregnant or HIV-positive after random confirmatory testing during their first ANC visit. Within the CCT intervention arm, 18 were removed from the study for similar reasons, including one woman who could not be followed-up for enrolment because she was admitted as an inpatient immediately after ANC registration and one who was a duplicate entry. Thus, 254 women in standard care and 263 women in the CCT intervention arm were included in the final ITT analysis. Another 68 women randomised to the intervention arm refused to enrol in the CCT programme and were thus excluded from the PP analysis. Among those who refused enrolment, 31.7% of women said that they needed their partner’s permission, 16.0% said they did not need the money, 13.4% did not want their photograph taken for biometric verification, 8.5% were struggling to comprehend their newly diagnosed HIV status, and 6.1% cited fear and stigma.

Table 2 displays the characteristics of women randomised to the CCT intervention and standard care arms. There were no significant differences in ages and month of pregnancy at the time of ANC registration between women in standard care and those in the CCT intervention arm. However, the distribution of participants recruited from different facilities was significantly different (*p* = 0.014): a higher percentage of women at Ikot Ekpene General Hospital were assigned to the CCT intervention arm (*n* = 98; 37.3%) than to standard care (*n* = 66; 26.0%), whereas a higher percentage of women at Eket General Hospital were assigned to standard care (*n* = 67; 26.4%) than to the CCT intervention (*n* = 51; 19.4%). As a result, we estimated the ITT and PP effects with and without controlling for recruitment site. Based on the additional data collected by the intervention administrators for women enroled in the CCT intervention, over 70% had at least one child, among which nearly 47% delivered their last child at a public or private health facility.

**Table 2:** Baseline characteristics of the intention-to-treat population

Characteristic	Standard care		Conditional cash transfer intervention		*P*-value
	N	Median or %	N	Median or %	
Recruitment site	254		263		
Ikot Ekpene	66	26.0%	98	37.3%	0.014
Oron	121	47.6%	114	43.3%	
Eket	67	26.4%	51	19.4%	
Age^a^	254	27.0	195	28.0	0.220
Month of pregnancy at ANC registration^a^	254	6.0	233	6.0	0.072
Marital status^b^			195		
Married			166	85.1%	
Single			24	12.3%	
Widowed			5	2.6%	
Number of children currently alive^b^			195		
0			50	25.6%	
1			64	32.8%	
2			42	21.5%	
3+			39	20.0%	
Place of delivery for last child born^b,c^			158		
Public hospital/clinic			68	43.0%	
Church			46	29.1%	
Traditional birthing center			25	15.8%	
Home			13	8.2%	
Private hospital/clinic			6	3.8%	
Newly diagnosed with HIV^b^			195		
Yes			68	34.9%	
No			127	65.1%	

^a^Age and month of pregnancy at ANC registration were not found for some women randomised to the intervention but who refused to enrol. Differences by study arm were tested using the Kruskal-Wallis non-parametric test

^b^Additional information on women enroled in the intervention were obtained from intervention administrators collected through surveys administered at enrolment and during follow-up phone calls with participants

^c^Further restricted to participants who have at least one child

Table 3 displays the estimated ITT effects for facility-based deliveries. Women offered the CCT programme were significantly more likely to give birth at a facility (41.4%) compared to women in standard PMTCT care (31.5%). This represents a statistically significant absolute difference of 9.3% (ORa = 1.50, 95% CI 1.04–2.16, *p* = 0.031) in adjusted estimates. When excluding women who declined to enrol in the CCT programme when offered, the absolute difference increased to 16.3% (ORa = 2.01, 95% CI 1.36–2.98, *p* = 0.000).

**Table 3:** Estimated effects of the conditional cash transfer programme on facility-based deliveries

	Total number of women (N)	Number who delivered at the facility (N)	Percent of babies delivered at the facility	
			Unadjusted	Adjusted
Intent-to-treat (ITT)				
Offered CCT intervention	263	109	41.4%	41.0%
Standard care	254	80	31.5%	31.9%
Risk difference percentage points (95% CI)			9.9 (1.7–18.2)	9.3 (0.9–17.7)
Odds ratio (95% CI)			1.54 (1.07–2.21)	1.50 (1.04–2.16)
Per protocol (PP)				
Enroled in CCT intervention	195	94	48.2%	47.9%
Standard care	254	80	31.5%	31.7%
Risk difference percentage points (95% CI)			16.7 (7.7–25.8)	16.3 (7.1–25.5)
Odds ratio (95% CI)			2.02 (1.38–2.98)	2.01 (1.36–2.98)

Results for EID testing are summarized in Table 4. For women with facility-delivered newborns due for EID testing, those offered the CCT programme were also more likely to have the baby tested (26.2%) than those in standard PMTCT care (13.4%). This translates into an ITT absolute difference of 12.4% (ORa = 2.24, 95% CI 1.42–3.53, *p* = 0.000) and a PP absolute difference of 18.7% (ORa = 3.06, 95% CI 1.90–4.91, *p* = 0.000).

**Table 4:** Estimated effects of the conditional cash transfer programme on newborn early infant diagnosis (EID) testing

			Percent of babies getting an EID test	
	Total number of babies in each group (N)	Number of babies tested (N)	Unadjusted	Adjusted
Intent-to-treat (ITT)				
Offered CCT intervention	263	69	26.2%	26.0%
Standard care	254	34	13.4%	13.6%
Risk difference percentage points (95% CI)			12.8 (6.1-19.6)	12.4 (5.6-19.2)
Odds ratio (95% CI)			2.30 (1.46-3.62)	2.24 (1.42-3.53)
Per protocol (PP)				
Enroled in CCT intervention	195	63	32.3%	32.1%
Standard care	254	34	13.4%	13.5%
Risk difference percentage points (95% CI)			18.9 (11.1-26.7)	18.7 (10.9-26.5)
Odds ratio (95% CI)			3.09 (1.93-4.94)	3.06 (1.90-4.91)

For the secondary outcome of nevirapine administration, displayed in Table 5, results were similar to those for facility delivery. Overall, 31.7% (164/516) of all participants had nevirapine given to their newborn as verified by hospital records—27.6% among those in standard care and 35.7% among those in the intervention. This unadjusted ITT absolute 8.2% difference (OR = 1.46, 95% CI 1.01–2.12, *p* = 0.046) is statistically significant, but not when adjusted for recruitment site (ORa = 1.39, 95% CI 0.96–2.03, p = 0.084). In PP estimates, the percentage of newborns given nevirapine increased to 43.6% within the intervention arm and the adjusted absolute 15.0% difference was statistically significant (ORa = 1.96, 95% CI 1.32–2.93, *p* = 0.001). Among facility-based births, the percentage of newborns receiving nevirapine was similar across arms: 87.5% (*n* = 70/80) among standard care mothers and 86.2% (*n* = 94/109) among CCT intervention mothers.

**Table 5:** Estimated effects of the conditional cash transfer programme on nevirapine administration of newborns delivered at the facility

	Total number of babies in each group (N)	Number of babies given nevirapine (N)	Percent of babies given nevirapine	
			Unadjusted	Adjusted
Intent-to-treat (ITT)				
Offered CCT intervention	263	94	35.7%	35.2%
Standard care	254	70	27.6%	28.1%
Risk difference percentage points (95% CI)			8.2 (0.2–16.2)	7.1 (−0.1–15.2)
Odds ratio (95% CI)			1.46 (1.01–2.12)	1.39 (0.96–2.03)
Per protocol (PP)				
Enroled in CCT intervention	195	85	43.6%	43.0%
Standard care	254	70	27.6%	28.0%
Risk difference percentage points (95% CI)			16.0 (7.2–24.9)	15.0 (6.1–23.9)
Odds ratio (95% CI)			2.03 (1.37–3.02)	1.96 (1.32–2.93)

**Fig. 2:**
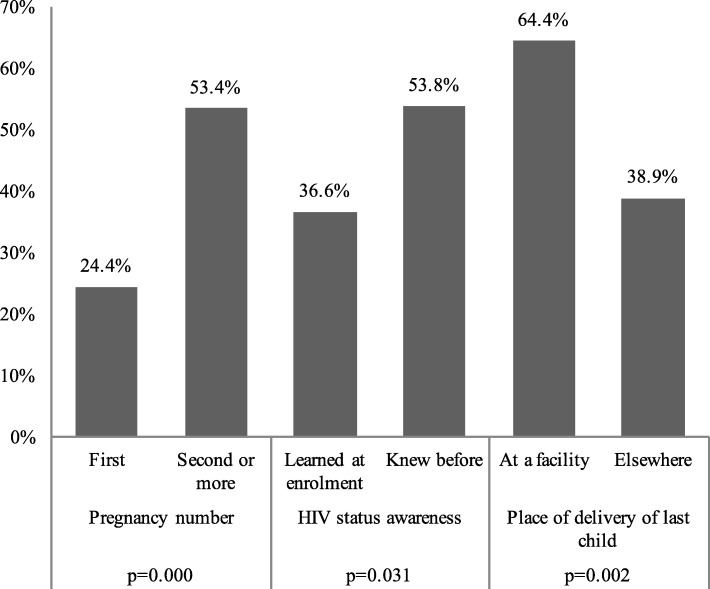
Percentage of babies delivered at the facility among women enroled in the conditional cash transfer programme. Notes: Differences tested using logistic regression controlling for recruitment site. Pregnancy number outcome restricted to mothers who have previously given birth

**Fig. 3:**
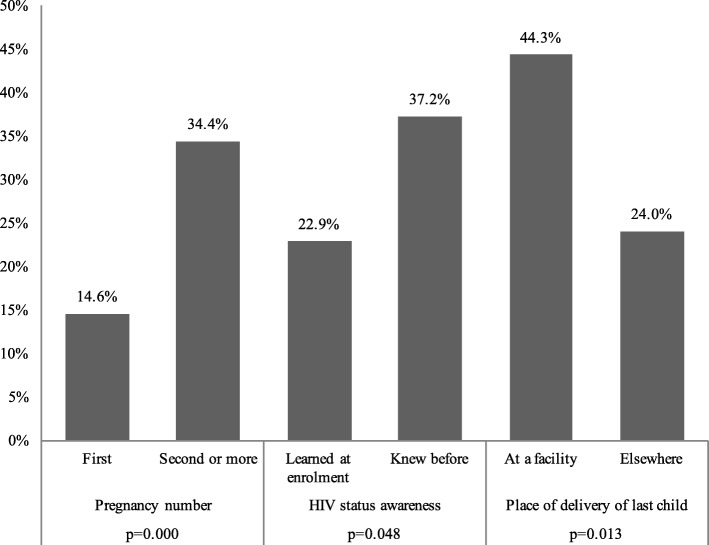
Percentage of facility-delivered babies with EID testing among women enroled in the conditional cash transfer programme. Notes: Differences tested using logistic regression controlling for recruitment site. Pregnancy number outcome restricted to mothers who have previously given birth

Among women offered the CCT programme, the effect on facility delivery differed by subgroups of beneficiaries (see Fig. 2), including those who had previously been diagnosed with HIV (53.8%) compared to those who were newly diagnosed the day of ANC enrolment (36.6%; *p* = 0.031). In addition, higher percentages of facility deliveries were observed for women who had previously been pregnant (53.4%) compared to women who were having their first child (24.4%; *p* = 0.000). Likewise women who delivered their last child at a facility were more likely to deliver in a facilty (64.4%) compared to those who delivered child elsewhere (38.9%; *p* = 0.002). Differences in EID testing followed the same pattern (Fig. 3).

## Discussion

Results from our evaluation of the CCT intervention showed that women enroled in the CCT intervention were more likely to deliver their babies at a facility (adjusted risk difference = 16.3 percentage points), were also more likely to have nevirapine administered to their infant at birth (adjusted risk difference = 15.0 percentage points), and were more likely to obtain an EID test (adjusted risk difference = 18.7 percentage points). All three outcomes were statistically significant in PP analyses; effect estimates were somewhat attentuated and less precise when including women who refused to enrol in the CCT intervention (ITT effects). Thus, we conclude that the intervention increased the uptake of two critically important steps in the PMTCT cascade when compared to standard PMTCT care without the incentives.

Our study contributes to the growing evidence on the effectiveness of CCTs for encouraging healthy behaviors. While a number of social-behavioral interventions for PMTCT have targeted various demand-side barriers, such as invitation cards to increase male involvement [31], couples counseling [32], and peer mentorship [33], only one other study has tested the impact of financial incentives (see below) [34]. Cash incentives, particularly those targeted to women, can additionally help to purchase health-promoting services and products. Indeed, among the women who received transfers and responded to survey questions in this study, most reported purchasing items for their newborn, including food, clothes, and toiletries (data not shown). CCT programmes have increasingly been incorporated into national health and education programmes in different countries [35, 36]. Even in Nigeria, CCTs for improving maternal and child health service utilization operated by the Federal Government have had substantial impact on ANC visits and preventive care during pregnancy, suggesting larger interest among policymakers in such an approach for strengthening social safety nets [37].

In the context of PMTCT, only one other study has examined the influence of CCTs for PMTCT. Yotebieng and colleagues found that very modest financial incentives (US$5, plus US$1 increment at every subsequent visit) also significantly increased retention in care among HIV-infected pregnant women in the Democratic Republic of Congo [34]. One major difference between their setting and ours is the background rate of facility delivery. More than 97% of births in Kinshasa Province, and nearly 80% of births in the Democratic Republic of Congo nationally, are delivered in a health facility [38], compared to fewer than 36% in Nigeria [18]. Hence, the design and impact of CCTs is in many ways shaped by the context in which it is being implemented.

It is also important to note that the observed effects of our CCT intervention may not reflect the impact of the cash transfers alone. Programme participants also received regular phone and SMS communications that may have also motivated them to remain in care. In particular, intervention administrators provided positive encouragement and support by way of advice, referrals, and reminders throughout the duration of the intervention period. These resources may be valued and engender behavior change, particularly in settings where HIV-related fear, concerns about ARV drug effects, and stigma are prevalent. Indeed, SMS and phone call reminders have increased uptake of HIV testing in Kenya [39] and increased rates of medical care for HIV-infected children in Cameroon [40]. Additional studies are needed to test the separate and interactive effects of these supportive resources from the cash incentives, and to assess the degree of complementarity in these approaches.

In our study, among those enroled in the CCT intervention, larger effects were also observed for women who were experiencing their first pregnancy and among women who had already known their HIV status prior to ANC registration. Anecdotes from intervention officers indicate that previously diagnosed women were often more receptive to the programme and understood that receiving the right medical treatment was important to reducing infection risk for their newborns. In contrast, newly diagnosed women sometimes denied their HIV status, which prevented them from wanting to be associated with the programme. Indeed, 15.5% of women who refused to enrol in the CCT intervention cited stigma, fear, and trouble comprehending their status as their reason for refusal. This result corroborates findings that show increased loss to follow-up among pregnant women who learn their status on the same day they begin treatment [41] and qualitative findings of women expressing fear of disclosing HIV status and stigma as reasons for lack of adherence [30]. Similarly, women having their first child may be more apt to undergo the recommended treatment to promote the health of their newborn compared to women who have already experienced a pregnancy and may already have beliefs about proper care. Our subgroup analysis also suggests that the CCT programme may have influenced the decision over where to deliver their child for at least some women: 36% of mothers who delivered their child at the facility while enroled in the CCT programme had delivered their previous child at a church, at home, or with a traditional birth attendant (data not shown).

### Limitations

These results should be interpreted in light of several caveats. Intervention administrators encountered several operational challenges that shaped the design of the CCT payouts, which in turn decrease the generalizability of the results. First, verification of completed steps was difficult given variability in record-keeping across facilities and across units within facilities, and the lack of reliable identifiers for patients. This resulted in an inability to track certain outcomes, such as ARV pickups. Women who may have delivered their child at facilities not included in the study were also unable to be tracked due to the lack of integrated information systems across facilities. Second, due to the formal and informal fees incurred by pregnant women, the size of the transfers and the lack of mobile banking in Nigeria required cash payouts be retrieved in person, placing additional time and monetary burden on participants to go to a bank, which may have negatively affected their interest in remaining in the intervention. Third, to accommodate the standard operating procedures for ANC enrolment, screening for eligibility prior to randomisation was not feasible or was surveying women in the control arm to collect more background characteristics at the time of ANC registration. Although exclusions due to ineligibility were similar across arms, we could not further assess the similarities of the two study arms beyond the few variables available in patient records (e.g., education, wealth, partner characteristics). Fourth, about one fourth of women randomised to be offered the CCT intervention declined to participate in the programme. While these refusals only affect PP estimates and not ITT estimates, and some self-selectivity may have helped increase the targeting of the intervention toward beneficiaries most in need (i.e., those who did not need the money did not participate), additional programme design features may want to expicitely address other factors that impeded initial uptake, including obtaining partner permission, biometric identification requirements, and dealing with a new HIV diagnosis. Lastly, some potentially eligible women may have been missed if they had come to facility for ANC care on a non-designated day; clinicians generally asked women to come back on the designated day, but some may have chosen not to do so.

Overall, our study results may not be generalizable beyond the study population in Akwa Ibom as the intervention programme features (e.g., size of cash transfers, verifiable incentivized conditions) were specifically tailored to what was operationally feasibile, likely to have impact within the local health system, and what the priorities of the patient population were. Further programme evaluation and/or replication is needed to understand how CCTs can be implemented, well-targeted, and scaled for broader reach and impact. For example, future CCT studies on PMTCT could test the effects of different cash transfer amounts that may be less burdensome to retrieve and easier to administer. With improvements in administrative data collection, future studies could also test the effect of incentives for transfers on ARV pick-ups at the hospital, steps in the cascade that are not exclusive to pregnant women, and thus potentially reach a larger pool of potential HIV-positive beneficiaries.

## Conclusions

While our study results suggest that CCTs can increase retention in care for PMTCT in Akwa Ibom, Nigeria, a sizable portion of women (nearly 60%) still did not opt to deliver at a facility or obtain an EID test for their infant after birth even when incentivized. These results add to the nascent literature on the effectiveness and feasibility of financial incentives for improving PMTCT utilization and retention in care. Financial incentives may be one promising strategy for reducing HIV infections among newborns through vertical transmission, but it is not a panacea and additional strategies are needed to further improve retention in care.

## Abbreviations

ANC: Antenatal care
ARV: Antiretroviral
ATM: Automatic teller machine
CCT: Conditional cash transfer
CI: Confidence interval
EID: Early infant diagnosis
HIV: Human immunodeficiency virus
IRB: Institutional Review Board
ITT: Intent-to-treat
NGO: Non-governmental organization
OR: Odds ratio
ORa: Adjusted odds ratio
PMTCT: Prevention of mother-to-child transmission
PP: Per Protocol
RCT: Randomised controlled trial

## Footnotes

1 Due to banking hours and anti-fraud measures, cash could not be disbursed the same day as intervention enrolment.

2 Regular review of hospital registsers included the following: Patient Card, Laboratory Investigation Report Form, Adult Pharmacy Worksheet, ARV Counselor’s worksheet, Daily HIV Test worksheet, Nurses’ notebooks, lab testers’ notebook, Delivery Register, Labor Ward Daily Report Book, Admissions Register, Child Follow-up Register, Dried Blood Spot Specimen Transport Form, and the Pediatric Pharmacy worksheet.

3 In addition to the intervention for this study, the implementing NGO operated other other CCT programmes in the same area; previous beneficiaries of CCT programmes implemented by the NGO outside this intervention were removed from this study, verified using biometric identification methods.

4 Of women randomized to the CCT intervention arm and who consented to enroling in the CCT programme, 12% were randomly selected for a HIV re-test and 7% were randomly selected for a pregnangy re-test. Random retesting was performed by the implementing NGO in order to reduce the likelihood of providers’ referring ineligible potential participants.

5 See https://www.dssresearch.com/KnowledgeCenter/toolkitcalculators/samplesizecalculators.aspx.

6 Only age and month of pregnancy at ANC enrolment are routinely recorded on facility patient registers. We could not further compare the comparability of our study arms because other background characteristics were not systematically available from facility medical records.
